# Expression of the human molecular chaperone domain Bri2 BRICHOS on a gram per liter scale with an *E. coli* fed-batch culture

**DOI:** 10.1186/s12934-021-01638-8

**Published:** 2021-07-30

**Authors:** Benjamin Schmuck, Gefei Chen, Josef Pelcman, Nina Kronqvist, Anna Rising, Jan Johansson

**Affiliations:** 1 Department of Biosciences and Nutrition, Karolinska Institutet, Neo, 141 86 Huddinge, Sweden; 2 Department of Anatomy, Physiology, and Biochemistry, Swedish University of Agricultural Sciences, Uppsala, Sweden

**Keywords:** Solubility tag, Bioreactor, High cell density culture, Protein expression, Protein purification

## Abstract

**Background:**

The human Bri2 BRICHOS domain inhibits amyloid formation and toxicity and could be used as a therapeutic agent against amyloid diseases. For translation into clinical use, large quantities of correctly folded recombinant human (rh) Bri2 BRICHOS are required. To increase the expression and solubility levels of rh Bri2 BRICHOS it was fused to NT*, a solubility tag derived from the N-terminal domain of a spider silk protein, which significantly increases expression levels and solubility of target proteins. To increase the expression levels even further and reach the g/L range, which is a prerequisite for an economical production on an industrial scale, we developed a fed-batch expression protocol for *Escherichia coli.*

**Results:**

A fed-batch production method for NT*-Bri2 BRICHOS was set up and systematically optimized. This gradual improvement resulted in expression levels of up to 18.8 g/L. Following expression, NT*-Bri2 BRICHOS was purified by chromatographic methods to a final yield of up to 6.5 g/L. After removal of the NT*-tag and separation into different oligomeric species, activity assays verified that different assembly states of the fed-batch produced rh Bri2 BRICHOS have the same ability to inhibit fibrillar and non-fibrillar protein aggregation as the reference protein isolated from shake flask cultures.

**Conclusions:**

The protocol developed in this work allows the production of large quantities of rh Bri2 BRICHOS using the solubility enhancing NT*-tag as a fusion partner, which is required to effectively conduct pre-clinical research.

**Supplementary Information:**

The online version contains supplementary material available at 10.1186/s12934-021-01638-8.

## Background

Alzheimer’s disease (AD) is the most common cause of dementia and belongs to a group of diseases that are characterized by the formation of deposits of specific proteins in amyloid fibrillar conformations [1]. Despite immense efforts from academia and the pharmaceutical industry, no disease-modifying treatment has been developed so far [2]. This is not only a problem for the patients and their families but also threatens to undermine the health care system at large since the progressive neurodegenerative nature of AD inevitably results in long-standing, severe decline in mental and physical status, and patients eventually require full-time attention [3]. This situation makes it necessary to investigate novel approaches to treat AD.

A large proportion of the human proteome has been found to contain amyloidogenic sequence motifs [4], suggesting that there are specific endogenous mechanisms that protect amyloidogenic proteins from forming amyloid that otherwise would cause disease. The BRICHOS domain (named from its discovery in Bri2, chondromodulin-1, and prosurfactant protein C) has emerged as the first example of an endogenous molecular chaperone-like domain that protects amyloidogenic protein segments from forming amyloid [5, 6]. The BRICHOS domains from different proteins can also prevent other amyloidogenic proteins than their natural targets from forming amyloid, and this raised the possibility to harness BRICHOS as a therapeutic strategy against amyloid diseases such as AD [7].

Mutations in the BRICHOS containing protein Bri2, which is associated with familial British and Danish dementias, give rise to the extended amyloidogenic peptides ABri and ADan, respectively [8, 9]. Bri2 BRICHOS can prevent amyloid formation also of non-client peptides, including amyloid β peptide (Aβ) and islet amyloid polypeptide (IAPP), associated with AD and type 2 diabetes, respectively [10, 11]. For instance, Bri2 BRICHOS prevents amyloid formation and neurotoxicity of the 42-residue Aβ42, the main amyloidogenic species in AD, in transgenic fruit flies and mice and prevents Aβ42 neurotoxicity in mouse hippocampal slices in vitro [12–15]. Moreover, recombinant human (rh) Bri2 BRICHOS passes the blood-brain barrier in wild-type mice, making parenteral administration of rh Bri2 BRICHOS an interesting new potential treatment against AD [16, 17]. Such application of rh Bri2 BRICHOS would require industrial protein production and purification protocols that result in a homogenous product at a reasonable cost.

Rh BRICHOS can be produced on a laboratory scale using strategies involving the use of fusion partners [18]. However, it was found that rh Bri2 BRICHOS forms different assembly states, primarily monomers, dimers, and high molecular weight oligomers consisting of about 20–30 subunits, which are difficult to resolve [15]. To circumvent this problem, we used a new production strategy that stems from insights into the silk production process of spiders.

Spider silk proteins (spidroins) are aggregation prone and have been found to form amyloid-like fibrils, making their production and storage at very high concentrations until silk formation takes place a formidable challenge [19]. The spidroin N-terminal domain (NT) works as a solubility enhancer that enables the storage of spidroins and prevents precocious aggregation [20]. This function can be harnessed also for improved solubility and the production of non-spidroins. In particular, a designed mutant, NT*, can keep very aggregation-prone proteins, including amyloid forming proteins, proteases, and membrane proteins, in solution [21–24]. NTs from widely different spidroins and spider species share the ability to improve solubility and increase recombinant production yields of aggregation prone proteins [23]. NT*, derived from the major ampullate spidroin from *Euprosthenops australis* spiders, was recently used as a tag to produce rh Bri2 BRICHOS and this enabled successful separation of the different assembly states [25]. Herein we investigate the feasibility to use *Escherichia coli* (*E. coli*) to produce NT*-Bri2 BRICHOS in bioreactors and study how the released target protein Bri2 BRICHOS acts as an inhibitor of fibrillar and non-fibrillar protein aggregation.

## Materials and methods

### Expression of NT*-Bri2 BRICHOS in *E. coli* bioreactor cultivations

A single colony of SHuffle T7 *E. coli* (New England Biolabs, Ipswich, USA) transformed with a pET-based plasmid encoding the gene for NT*-Bri2 BRICHOS [25] was used to inoculate 20 mL LB-medium (containing 50 mg/L kanamycin and 1% glycerol). The culture was incubated at 30 °C and 220 rpm in a baffled flask overnight. Then, the culture was diluted 50 to 100 times into fresh medium to obtain an OD_600_ of 0.05. In total 11 bioreactor cultivations were carried out in a Multifors 2 (Infors HT Basel, Switzerland) (Culture #1 to #5) or Minifors 2 (Infors HT, Basel, Switzerland) (Culture #6 to #11) with culture sizes in the range between 250–400 or 900–1200 mL, respectively. The pH of all cultures was continuously adjusted to 7, by the addition of either 3 M H_3_PO_4_ or 25% NH_3_. The oxygenation level, pO2 was set to 30%, which was achieved by increasing the stirrer speed and/or the airflow rate. All cultivation media described hereafter were adopted from earlier reported recipes and were prepared by first autoclaving the main components in the reaction vessel, followed by separate addition of sterile stock solutions of filtered glucose (700 g/L) and kanamycin (30 g/L), as well as autoclaved antifoam 204 (10%, Sigma Aldrich), trace metal solution (25×), and MgSO_4_ (500 g/L), where applicable. The kanamycin concentration was 30 mg/L in all cultivations, and the initial concentration of antifoam 204 was 0.01%.

In cultures #1 & 2, the expression of NT*-Bri2 BRICHOS using complex media formulations including Lysogeny Broth (LB) and Terrific Broth (TB) were tested. These were grown continuously at 30 °C in a batch manner and were induced by adding IPTG to 500 µM when OD_600_ was still below 1. As a next step, the expression levels of NT*-Bri2 BRICHOS in semi-defined and defined media according to da Silva [26] (Batch medium adapted from experiment #4, feed medium from experiment #3 without lactose), Korz [27], and Wyre [28] (semi-defined medium described in the paper) was tested (culture # 3–5). After growing the cultures to OD_600_ ≈ 30 (da Silva and Wyre) or 13 (Korz), they were induced by the addition of 33 µM IPTG. During the entire cultivation time, the temperature was held constant at 25 °C. In the next cultivation series, the medium formulation suggested by da Silva was used to identify the most optimal IPTG concentration to maximize the yield of soluble rh NT*-Bri2 BRICHOS. In this experiment, cultures #6–8 were grown at a constant temperature of 25 °C and induced with 50, 150, or 500 µM IPTG once OD_600_ reached ≈ 30 (≈1 L sized cultivations). Culture #9-11 is a replication of culture #7 (induction with 150 µM ITPG), with the exception that induction was implemented once OD_600_ reached 60. Furthermore, ~ 3 h before induction the temperature was carefully increased to 27 °C, to enable faster growth, and then reduced to 25 °C when the cultures were induced with IPTG:

Independent of the induction point, exponential feeding was initialized in all experiments once pO_2_ levels increased suddenly, indicating that the carbon source contained in the batch medium was depleted. The feeding rate was adjusted to an exponential feeding profile according to Eq. (1) [29]:

1 $$F=\frac{1}{S}*\left(\frac{\mu }{{Y}_{X/S}}+m\right)*{X}_{0}*{e}^{\mu t}$$

where *F* is the rate of feeding (L h^−1^), *s* the concentration of the glycerol/glucose in the feed (g L^−1^), *µ* the specific growth rate (h^−1^), *Y*_*X/S*_ the biomass yield on the substrate (g g^−1^), *m* the specific maintenance coefficient (g g^−1^ h^−1^), *X* the biomass concentration (g L^−1^). The specific growth rate *µ* was set to 0.1, *m* to 0.025 g g^−1^ h^−1^, and *Y*_*X/S*_ was 0.622 g g^−1^ [26]. A summary of all essential cultivation parameters is found in Table 1.

**Table 1:** Summary of key parameters for the expression and purification of NT*Bri2 BRICHOS expressed in bioreactor cultivations in this study

Culture	#1	#2	#3	#4	#5	#6	#7	#8	#9^d^
Temperature (°C)	30	30	25	25	25	25	25	25	25
Medium	LB	TB	da Silva	Korz	Wyre	da Silva	da Silva	da Silva	da Silva
Substrate in feed			Glycerol	Glycerol	Glycerol	Glycerol	Glycerol	Glycerol	Glycerol
IPTG (µM)	500	500	33	33	33	50	150	500	150
OD_600_ induced	1	0.6	30	13	35	32	25	29	60
OD_600_ harvest	8.4	31	121	50	61	190	106	76	176
Induction time (h)	21	21	15	10	10	20	20	20	22
Total culture time (h)	26	26	39	39	39	47	47	47	49
Culture Size (mL)	400	400	325	274	307	910	900	907	1180
Wet Cell Weight (g/L)	13	32	197	58	81	200	120	82	214^e^
Expression level (g/L)^a^	0.53	0.82	2.1	2.1	2.7	7.9	7.9	6.2	18.8^f^
% of culture purified	100%	100%	25%	100%	50%	9.1%	11.6%	25%	7.1%
Purified (mg)^b^	37	73	40	70	92	133	428	510	540
Yield after purification (g/L)^c^	0.09	0.18	0.49	0.26	0.60	1.61	4.12	2.25	6.41
% of expression level	17.6%	22.3%	23.8%	11.9%	22.1%	20.4%	51.8%	36.4%	34.1%

^a^Expression level of NT*-Bri2 BRICHOS was estimated with SDS-PAGE. See also Additional file 1: Figures S2, S3

^b^Protein yield was calculated using Abs_280_ and the protein specific extinction coefficient after NT*-Bri2 BRICHOS was eluted and dialyzed to remove imidazole

^c^Yield of NT*-Bri2 BRICHOS after purification

^d^Culture #9 was repeated 2 more times to estimate the batch-to-batch variation see Additional file 1: Table S1

^e^Dry cell weight for culture #9 was 82 g/L

^f^The expression level calculated by considering the % of NT*-Bri2 BRICHOS relative to the total protein content and the dry cell mass is 15.2 g/L

After cultivation, the cells were harvested in a Sorvall LYNX 6000 (Thermo Scientific) centrifuge, using an F9-6 × 1000 LEX rotor at 4000×*g* for 20 min. Then, the cell pellet was re-suspended by gentle agitation at 4 °C in Buffer A (20 mM tris, pH 8.0), using 20 mL buffer per 10 g wet cell weight. Cell resuspensions were stored at −20 °C.

### Purification using immobilized metal ion affinity chromatography (IMAC)

A 50 mL cell resuspension was thawed and lysed using a cell disruptor (Constant Systems, Daventry, United Kingdom) at 1.35 kbar. Then DNAse I (Roche, Basel, Switzerland) was added to the lysed cells to a final concentration of 10 µg/mL, followed by incubation on ice for 30 min, and by centrifugation at 38,000×*g* in a Sorvall RC 6 + (Thermo Fisher Scientific) using an F21-8 × 50y rotor (4 °C for 30 min). After centrifugation, the supernatant was filtered through a Filtropur S 0.45 (Sarstedt, Nümbrecht, Germany). All subsequent purification steps were carried out on an Äkta Explorer liquid chromatographic system (GE Healthcare, Uppsala, Sweden) at 6 °C. The lysate was loaded onto 4 × 5 mL HiTrap Chelating HP columns (GE Healthcare, Uppsala, Sweden) equilibrated with buffer A using a flow rate of 2.5 mL/min. Next, the proteins bound to the column were washed with buffer A containing 40 mM imidazole, before NT*-Bri2 BRICHOS was eluted with buffer A containing 200 mM imidazole. After elution, the flow-through was re-applied onto the columns, and NT*-Bri2 BRICHOS was washed and eluted in the same manner one more time. The combined eluate was subsequently dialyzed against 5 L buffer A overnight using a Spectra Por membrane (Spectrum Labs, Rancho Dominguez, USA) with a molecular weight cut-off between 6 and 8 kDa. All samples were stored at −20 °C.

### Estimation of the NT*-Bri2 BRICHOS expression level and the yield after purification

To estimate the expression levels of NT*-Bri2 BRICHOS in the different cultures before harvest, a 100 µL sample (undiluted culture #1; threefold diluted culture #4; fivefold diluted culture #5; tenfold diluted culture #3, and #6–9; 20-fold diluted culture #10–11) was centrifuged to pellet the cells. The supernatant was discarded, and the pellet was re-suspended into 100 µl 8 M urea. 10 µL of each sample was loaded onto a 4–20% Mini-protean TGX stain-free precast SDS-PAGE gel (Bio-Rad, Munich, Germany). To estimate the amount of protein in the samples, already purified NT*-Bri2 BRICHOS was used as standard: 0.24, 0.48, 0.95, and 1.9 mg/ml unless noted otherwise. To create a standard curve, the standard bands were integrated with ImageLab software (Biorad, Munich, Germany), which correlated the band intensity to protein concentration.

The concentration of purified NT*-Bri2 BRICHOS was calculated using the protein specific extinction coefficient, Abs 0.1% (= 1 g/L) = 0.492, after measuring the absorbance at 280 nm in triplicates. This method was also used to determine the concentration of the NT*-Bri2 BRICHOS standards used to estimate the expression level with SDS-PAGE.

The dry cell mass of culture #9–11 was determined by transferring 1 mL cell resuspensions (in triplicates), to already dried and weighed Eppendorf tubes. The cells were pelleted by centrifugation, the supernatant was removed, and the cell paste was dried at 65 °C for at least 24 h until a stable weight was obtained. This method enabled an alternative way to estimate the expression level of culture #9-11, by determining the % of NT*-Bri2 BRICHOS relative to the total cellular proteins, using GelAnalyzer 19.1, and considering that the protein fraction of the total dry cell weight is 55% [30].

### Isolation of the different oligomeric species using size exclusion chromatography

IMAC (immobilized metal affinity chromatography) purified NT*-Bri2 BRICHOS was concentrated to 3.4 mg/mL with a Vivaspin protein concentrator (10 kDa cut-off, GE Healthcare) at 4000×*g* and 4 °C. Then, the different rh NT*-Bri2 BRICHOS oligomeric species were isolated by size exclusion chromatography (SEC) using a Superdex 200 26/600 column (GE Healthcare, Uppsala, Sweden), which was equilibrated with Buffer B (20 mM NaP_i_ buffer pH 8.0 containing 0.2 mM EDTA), and an ÄKTA Explorer liquid chromatographic system (GE Healthcare, Uppsala, Sweden). The individual peaks corresponding to the differently sized oligomeric species were narrowly collected. Then, the rh NT*-Bri2 BRICHOS species were cleaved separately with 1: 600 thrombin (w/w, Merck) in a cold room overnight. After cleavage, the NT* tag was removed by reapplying the sample onto an IMAC column with buffer B (reverse IMAC), where rh Bri2 BRICHOS was found in the flow-through, and the NT*-tag bound to the column was eluted with buffer B containing 200 mM imidazol. To polish the Bri2 BRICHOS species, they were further purified with SEC one more time, using a Superdex 200 26/600 (for oligomer, tetramer, and dimer), or a Superdex 75 26/600 (for monomer) column (GE Healthcare, Uppsala, Sweden). The purified Bri2-BRICHOS species were stored at −20 °C.

### Citrate synthase thermal aggregation

The ability of the different rh Bri2 BRICHOS species to prevent non-fibrillar protein aggregation was assessed by suppressing thermo-denaturation of citrate synthase (CS). CS from porcine heart (Sigma-Aldrich, Germany) was diluted in 40 mM HEPES/KOH pH 7.5 to 600 nM and then incubated at 45 °C with and without different concentrations of rh Bri2 BRICHOS oligomer or tetramer. The aggregation kinetics were monitored in triplicate using a microplate reader (FLUOStar Galaxy from BMG Labtech, Offenberg, Germany) by reading the apparent absorbance at 360 nm, as a measure of turbidity, under quiescent conditions.

### Aβ42 monomer preparation and Thioflavin T assay

Aβ42 fused with a solubility tag (NT*) was expressed in BL21*(DE3) pLysS *E. coli* (B strain) cells (Novagen) and purified as described previously [23]. Briefly, NT*-Aβ42 was purified by IMAC, and the NT* was released by TEV cleavage (1:100, w/w, in-house recombinantly prepared). The crude cleaved sample was lyophilized and re-dissolved in 20 mM Tris pH 8.0 containing 7 M Gdn-HCl. Pure Aβ42 monomers were isolated with a Superdex 75 column (GE Healthcare, Uppsala, Sweden) using a 20 mM sodium phosphate pH 8.0 buffer containing 0.2 mM EDTA. The Aβ42 monomer concentration was calculated with a protein specific extinction coefficient of 1 424 M^−1^ cm^−1^ for (A_280_-A_300_).

For testing the activities of rh Bri2 BRICHOS species against Aβ42 fibril formation, 80 µL reaction solution containing 3 µM Aβ42 monomers, 10 µM Thioflavin T (ThT) and different concentrations of Bri2 BRICHOS species at molar ratios 0, 10, 50, and 100% relative to Aβ42 were added to each well of half-area 96-well microplates (Corning Glass 3881, USA). The fluorescence was recorded using a 440 nm excitation filter and a 480 nm emission filter (FLUOStar Galaxy from BMG Labtech, Offenberg, Germany) under quiescent conditions at 37 °C. Aggregation traces are averages of four replicates for all the experiments. For comparing the activities of each Bri2 BRICHOS species, the aggregation half time ${\tau }_{1/2}$ and the maximal growth rate ${r}_{max}$ were extracted by an empirical sigmoidal Eq. (2) [25, 31]:

2 $$F={F}_{0}+A/(1+\mathrm{exp}[{r}_{max} ({\tau }_{1/2}-t)] )$$

where *A* is the amplitude and *F*_*0*_ the base value.

### Stability of Bri2 BRICHOS oligomers and dimers

Rh Bri2 BRICHOS oligomers (10 μM) and monomers (5 μM) expressed in the bioreactor were incubated in 20 mM sodium phosphate pH 8.0 with 0.2 mM EDTA overnight at 37℃, and samples were analyzed by SDS-PAGE under native, reducing, and non-reducing conditions.

## Results

### Expression and purification of NT*-Bri2 BRICHOS in the bioreactor

A typical yield of NT*-Bri2 BRICHOS is 80 mg/L when purified with IMAC after expression in a shake flask type cultivation, using LB medium and inducing with 500 µM IPTG when the OD_600_ is below 1. This is comparable to the yield of purified NT*-Bri2 BRICHOS produced by bioreactor culture #1, which essentially follows this simple shake flask expression protocol (a summary of all the essential parameters of each culture is summarized in Table 1). Notably, the same expression protocol, but using the richer TB medium doubled the yield to 180 mg/L (culture #2). Because we did not expect any significant improvement by optimizing the expression protocol using these complex media in terms of the cell density, we tested more sophisticated formulations, which included two semi-defined and one defined medium, originally described by da Silva, Korz, and Wyre (Culture #3, #4, and #5, respectively) [26–28]. Since native Bri2 BRICHOS has one disulfide bond and tends to form higher-order assemblies [25], we proceeded with a cultivation strategy that minimizes the cellular stress level to maximize the expression levels [28, 32]. Thus, instead of fast growth at 37 °C before induction, we aimed to keep the temperature at a constant level of 25 °C throughout the entire cultivation. Furthermore, it is known that induction with high IPTG concentrations inhibits the exponential growth of *E. coil* [33]*,* and therefore we induced with only 33 µM IPTG in the subsequent cultures.

**Fig. 1:**
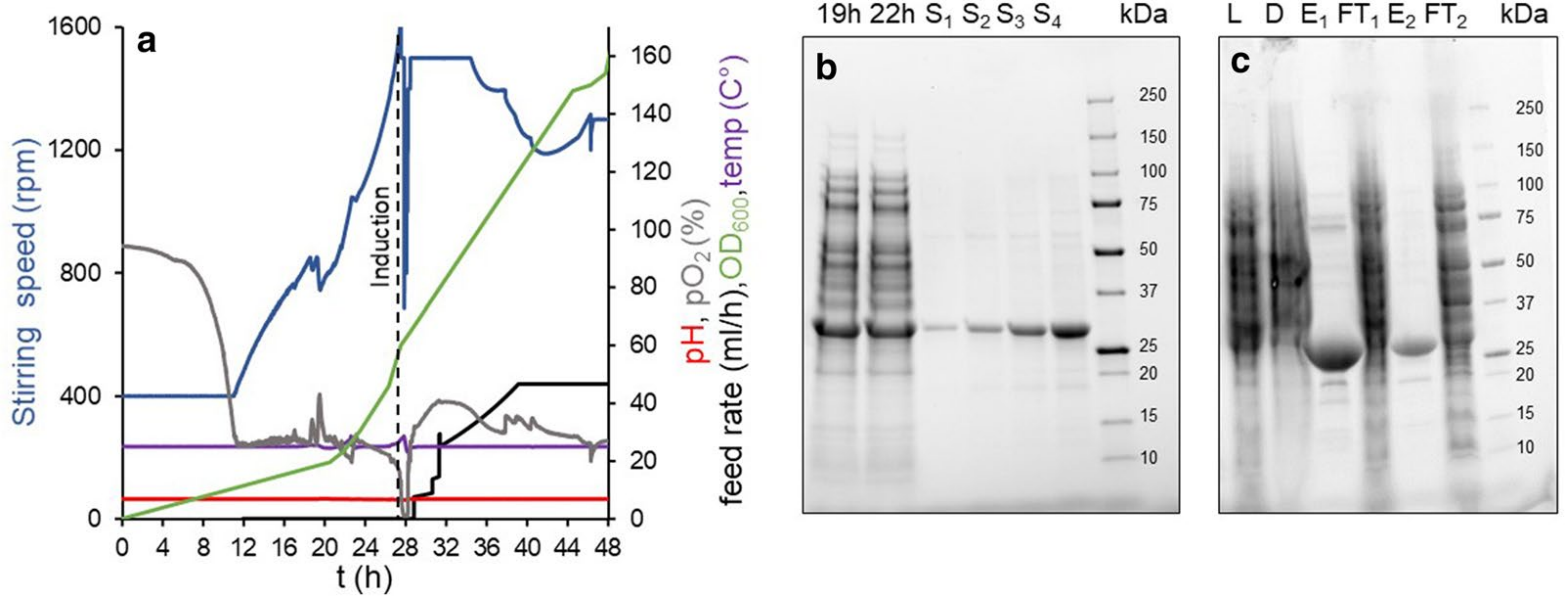
Bioreactor production of rh Bri2 BRICHOS. **a** Fed-batch cultivation (Culture #9). After 28 h, the oxygen demand suddenly decreased, and the feeding was initiated. **b** Estimation of expression level by SDS-PAGE. A sample from the cultivation 19 h and 21 h after induction was diluted 10-times. S1–S4 served as standards to estimate the concentration of NT*-Bri2 BRICHOS. S_1_ 0.24 mg/mL; S_2_ 0.48 mg/mL; S_3_ 0.95 mg/mL; S_4_ 1.9 mg/mL. **c** IMAC purification. L: cell lysate; D: pellet after centrifugation; E1: IMAC eluate; FT1: Flow-through round 1; E2: IMAC eluate after re-loading FT1; FT2: Flow-through round 2

Reaching OD_600_ 1.2 after 17 h at 25 °C, cells cultivated in the defined medium suggested by Korz exhibited the slowest growth in direct comparison to the semi-defined formulations by Wyre and da Silva (OD_600_ was 3.6 and 11.6, respectively). After a total culture time of 39 h, the da Silva-medium yielded an OD_600_ of 120, Wyre-medium an OD_600_ of 61, and Korz-medium an OD_600_ of 50. Even though the medium according to da Silva yielded the best results in terms of cell density, after purification the yield was 0.6 g/L soluble NT*-Bri2 BRICHOS for Wyre and 0.5 g/L for da Silva medium (Table 1). This is an improvement of the NT*-Bri2 BRICHOS yield by a factor of two compared to the preceding cultivations. However, substantial impurities were seen for the NT*-Bri2 BRICHOS produced using the da Silva medium (Additional file 1: Figure S1). Nevertheless, we decided to express NT*-Bri2 BRICHOS one more time in medium from da Silva, due to its high potential for dense microbial growth. To test whether the expression level could be improved further, we induced with 50, 150, and 500 µM IPTG once OD_600_ reached 30 (culture #6, #7, and #8, respectively). According to the OD_600_ measured before culture harvest and the wet cell mass after harvest, there is a possible negative correlation between the amount of IPTG used to induce the culture and the final cell mass, however, the expression levels of NT*-Bri2 BRICHOS were comparable (Table 1). Induction with 150 µM IPTG resulted in an expression level of 7.9 g/L, whereas the expression levels of the cultures induced 50 µM and 500 µM IPTG were 7.9 and 6.2 g/L, respectively. Considering that the highest protein yield after purification (4.1 g/L) was achieved with the culture induced with 150 µM IPTG, we attempted to improve this further, by changing the point of induction. Repeating the protocol of culture #7 but inducing expression at OD_600_ 60 instead of around 30 increased the expression level additionally by a factor of two to 18.8 g/L (culture #9, Fig. 1). Purification of NT*-Bri2 BRICHOS from this batch using IMAC yielded 6.4 g/L, which is the highest achieved in this study. Finally, to assess the reproducibility of this process, two more cultivations were performed replicating the conditions of culture #9. This time, the expression level was 10.3 g/L (culture #10) and 6.5 g/L (culture #11), which yielded 3.45 and 2.25 g/L of NT*-Bri2 BRICHOS after purification (Additional file 1: Table S1).

### Isolation of monomer, dimer, and oligomeric species of NT*-Bri2 BRICHOS

**Fig. 2:**
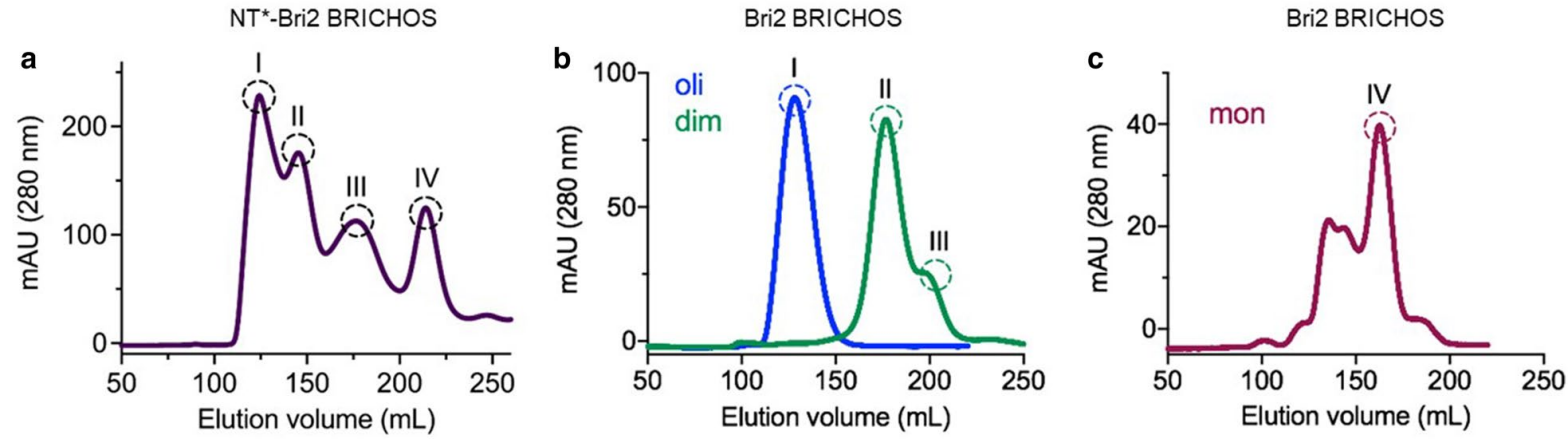
Size exclusion chromatography of NT*-Bri2 BRICHOS and Bri2 BRICHOS after cleavage with thrombin and reverse IMAC. **a** Chromatogram of NT*-Bri2 BRICHOS using a Superdex 200 column to isolate the individual oligomeric species (monomer: peak IV, dimer: peak III, tetramer: peak II and higher oligomers: peak I). The individual species were then cleaved with thrombin to remove the NT*-tag and Bri2 BRICHOS was separated from the NT*-tag using reverse IMAC. **b** Chromatograms of oligomeric (I), tetrameric (II) and dimeric (III) Bri2 BRICHOS using a Superdex 200 column. **c** Chromatogram of monomeric (IV) Bri2 BRICHOS using a Superdex 75 column

**Fig. 3:**
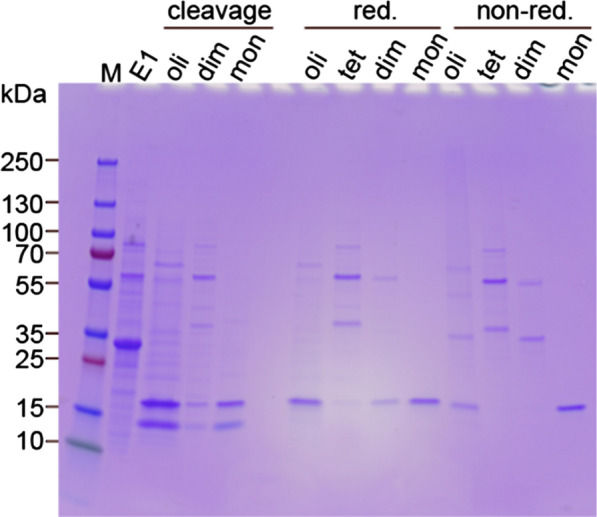
SDS-PAGE analysis of the rh Bri2 BRICHOS species isolated with size exclusion chromatography. E1 indicates the eluted NT*-Bri2 BRICHOS from IMAC. Oligomer (oli), dimer (dim) and monomer (mon) fractions labeled with ‘cleavage’, correspond to the SEC isolated NT*-Bri2 species after overnight thrombin cleavage. After individual cleavage, each species was purified with reverse IMAC to remove the NT*-tag, followed by SEC, to isolate the oligomer, tetramer, dimer, and monomers. The individual species were collected and analyzed by SDS-PAGE under reducing (red.) and non-reducing (non-red.) conditions. M indicates the protein ladder. Noteworthy, the tetramer (tet.) fraction also contains a small amount of hexamer, which could have formed after collection of the corresponding peak eluted from SEC. The dimer band visible in the same fraction could either be a contamination from the adjacent dimer peak or emanate from a non-covalent tetramer

NT*-Bri2 BRICHOS is prone to form different oligomeric species, that differ with respect to their potency to inhibit amyloid formation and non-fibrillar protein aggregation, respectively [25]. To enable a more precise investigation of NT*-Bri2 BRICHOS produced in the bioreactor, the protein was separated into monomers, dimers, and higher order oligomers with size exclusion chromatography (Fig. 2a). From this experiment interestingly four peaks were resolved, whereas three peaks are isolated from NT*-Bri2 BRICHOS expressed in shake flasks, corresponding to peaks I, III, and IV in Fig. 2a. The additional peak (II) corresponds to a tetramer (Fig. 3). The individual NT*-Bri2 BRICHOS species were cleaved with thrombin and the rh Bri2 BRICHOS species were separated from the NT*-tag (containing a His_6_-tag) by reverse IMAC. Finally, the rh Bri2 BRICHOS species were concentrated before isolation with SEC to obtain pure oligomeric, tetrameric, dimeric, and monomeric species (Fig. 2b, C) and their prolonged stability at 37 °C was confirmed (Additional file 1: Figure S4).

### Inhibition of amyloid fibril formation and non-fibrillar protein aggregation

**Fig. 4:**
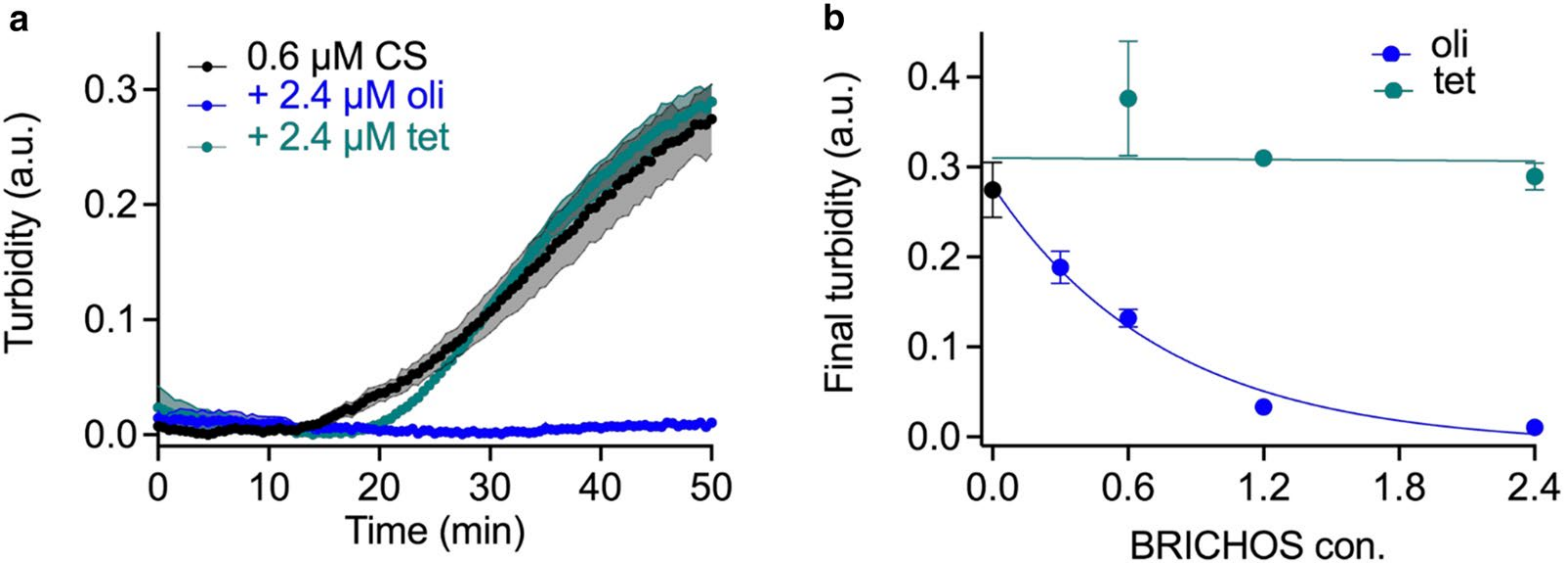
Activities of rh Bri2 BRICHOS oligomers and tetramers against citrate synthase in a turbidity assay. **a** Oligomeric (oli) species inhibit citrate synthase aggregation, whereas this effect is not seen for tetramers (tet). **b** Concentration dependent effect of rh Bri2 BRICHOS oligomers and tetramers on citrate synthase aggregation

To assess if differences exist between rh Bri2 BRICHOS expressed in the shake flask or using a bioreactor, the inhibitory effect of rh Bri2 BRICHOS against non-fibrillar aggregation of heated citrate synthase and amyloid fibril formation of Aβ42 was tested. In the assay involving citrate synthase, rh Bri2 BRICHOS oligomers showed a dose dependent activity against non-fibrillar protein aggregation and at a ratio of 1:2 (0.6 µM citrate-synthase vs 1.2 µM BRICHOS), the aggregation was completely inhibited (Fig. 4), similar to the capacity of the Bri2 BRICHOS oligomers produced in shake flasks [25]. The rh Bri2 BRICHOS tetramers did not show any inhibitory effects on thermo-induced citrate synthase aggregation, comparable to the lack of activity seen for monomer and dimer obtained from shake-flask expressed rh Bri2 BRICHOS [25]. These observations support that Bri2 BRICHOS large oligomers are required for general chaperone activity against amorphous protein aggregation.

**Fig. 5:**
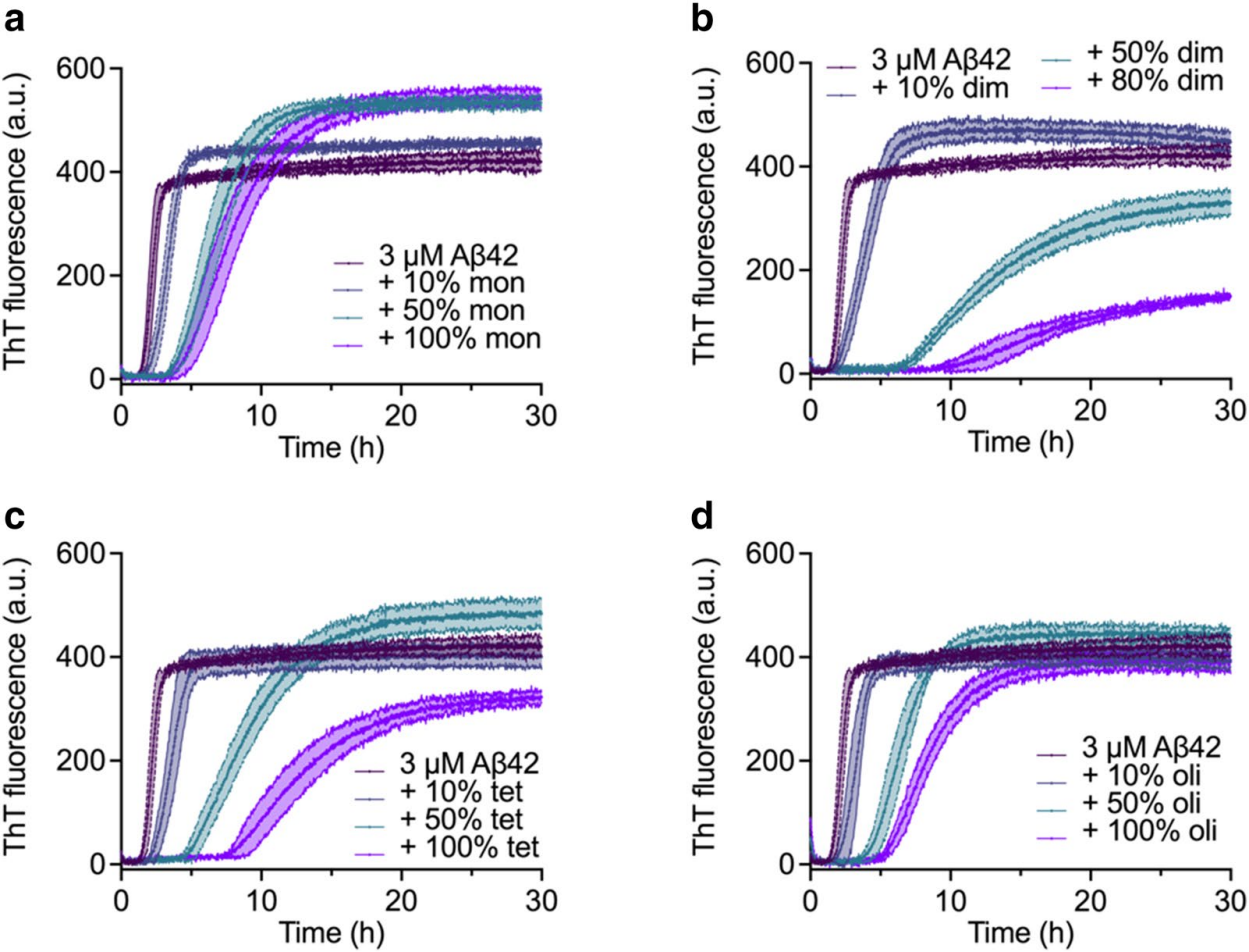
Time dependent fluorescence traces of Thioflavin-T to test the inhibition efficiency of rh Bri2 BRICHOS monomers (**a**), dimers (**b**), tetramers (**c**), and higher order oligomers (**d**) against fibril formation of Aβ42

**Fig. 6:**
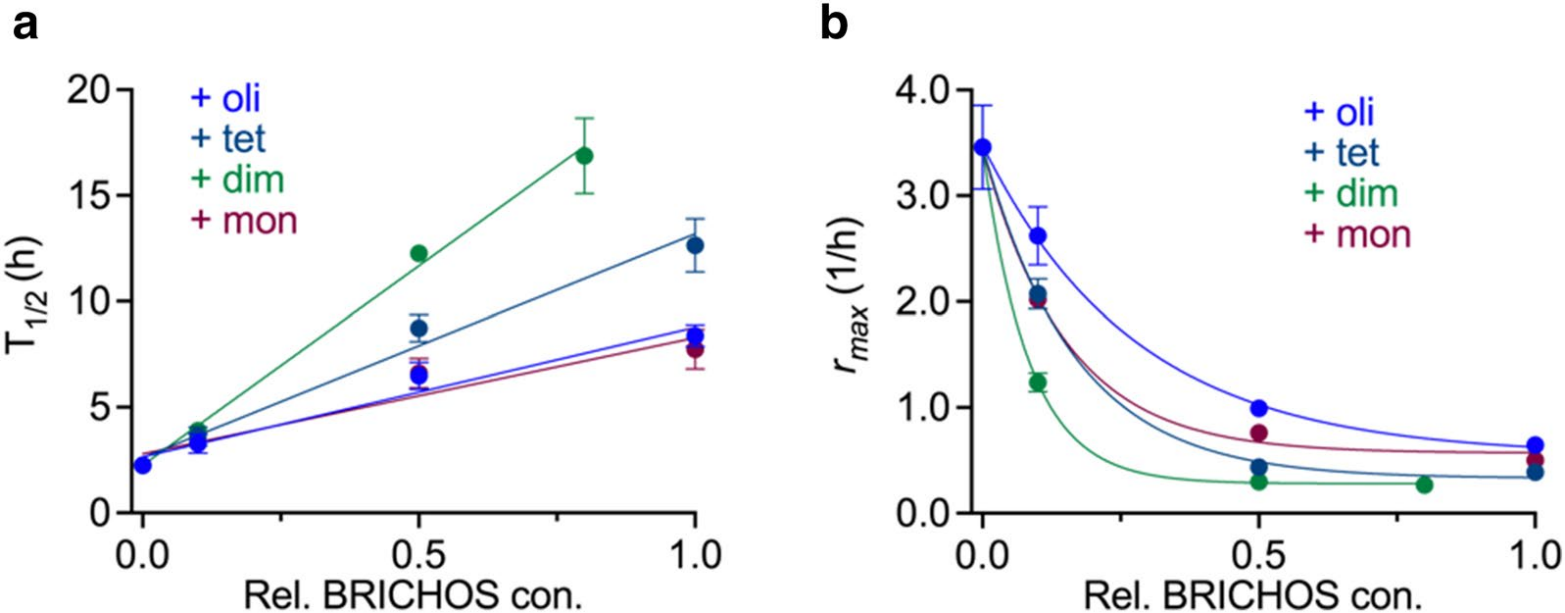
The aggregation half time ${\tau }_{1/2}$ (**a**) and the maximal growth rate ${r}_{max}$ (**b**) of rh Bri2 BRICHOS species against Aβ42 fibril formation

**Fig. 7:**
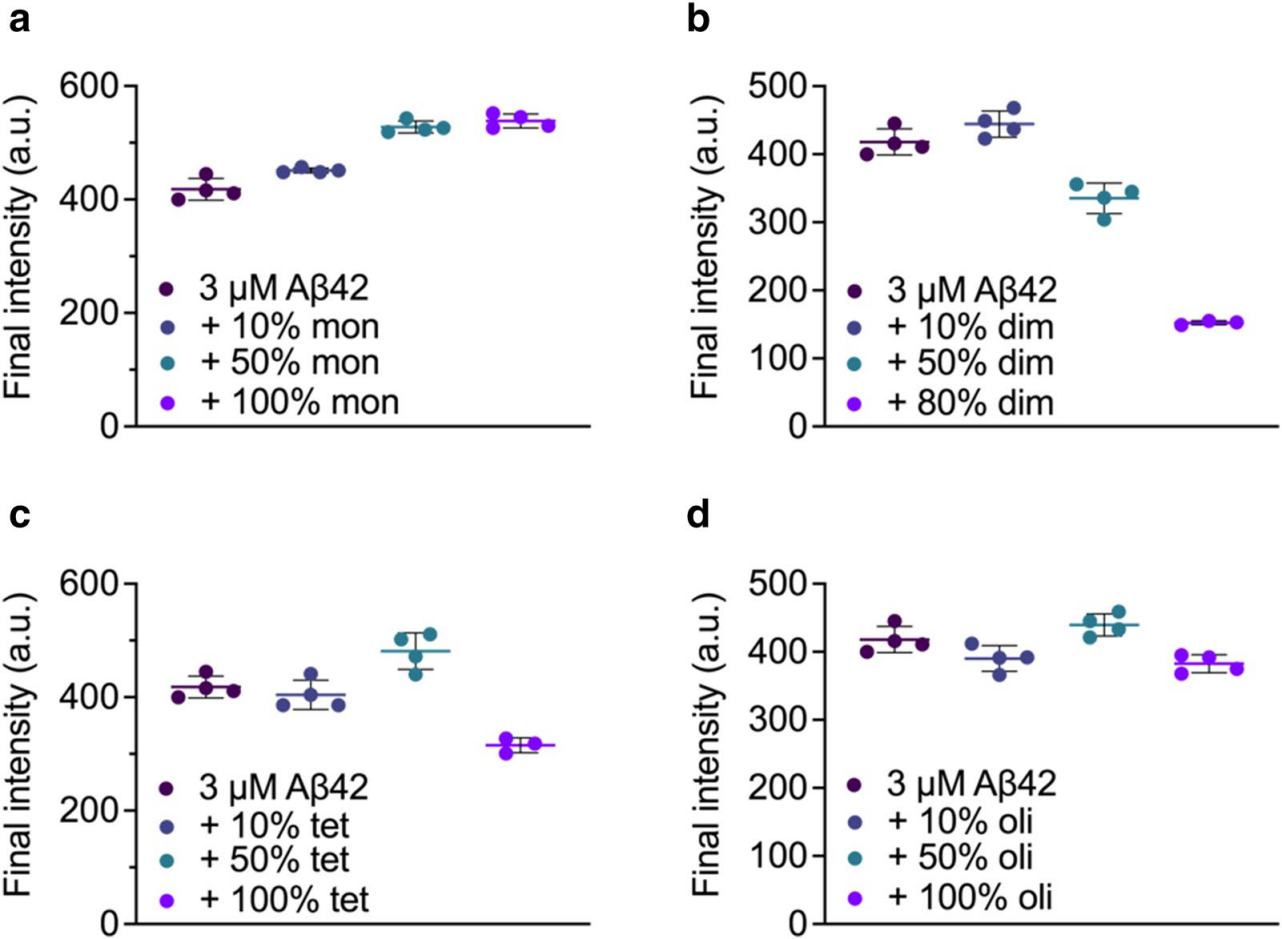
Final intensity of aggregation traces of the rh Bri2 BRICHOS monomers (**a**), dimers (**b**), tetramers (**c**), and higher order oligomers (**d**) against Aβ42 fibril formation

Next, the activities of different species against Aβ42 fibril formation were assessed using a ThT based assay. The different rh Bri2 BRICHOS species showed a dose-dependent influence on Aβ42 fibrillation traces indicated by ThT fluorescence (Fig. 5). Among them, the dimer and tetramer were the most efficient species, in particular the dimer, as they showed the most prominent effect on increasing ${\tau }_{1/2}$ (time until half maximum fluorescence is reached) and decreasing ${r}_{max}$ (the maximum growth rate) (Fig. 6). Further, the dimeric rh Bri2 BRICHOS lowered the final fluorescence intensity drastically, whereas the tetramer only showed minor effects on the final intensity at the highest concentration tested (Fig. 7). The effects on Aβ42 fibril formation and final intensity for the tetramer may in part be due to the presence of dimers in the tetramer fraction. The oligomers and monomers did not reduce the final ThT intensity. Thus, according to the increase in ${\tau }_{1/2}$, the reduction of maximum rate and the final ThT intensity, the relative inhibition efficiencies against Aβ42 fibril formation are dimer > tetramer > monomer > oligomer. Our previous data on the rh Bri2 BRICHOS species produced in shake flasks [25], in terms of activity against Aβ42 fibril formation, showed that the dimer had a ${\tau }_{1/2}$ of ~ 12 h, while ~ 10.5 h and ~ 5.5 h was achieved for the monomer and oligomer, respectively, giving the following efficiency order dimer > monomer > oligomer. Compared to the rh Bri2 BRICHOS species from shake flask cultures, the bioreactor produced rh Bri2 BRICHOS species thus showed similar or better efficiency in inhibition of Aβ42 fibril formation.

## Discussion

To use recombinant proteins for medical or biotechnological applications, the production needs to be scalable and the typical expression levels need to be in the high g/L scale to be economical [34, 35]. *E. coli* is a favorite workhorse for the production of biopharmaceuticals and is used for the production of about 30% of all clinically approved proteins [36], but a frequently occurring drawback of this heterologous host is the formation of inclusion bodies [28, 35, 37, 38]. To increase the yield and improve the solubility of otherwise aggregation prone peptides, the NT* domain was developed. NT* is a solubility tag that has successfully been used to increase the yield of several proteins up to a factor of eight, compared to other commonly used solubility tags [21, 23]. These results were obtained with shake flask cultivations using *E. coli*, hence the next logical step was to employ the NT*-domain for protein expression in a high-cell density bioreactor cultivation. In this study, we attempted to express and purify the rh Bri2 BRICHOS domain on a g/L scale employing the NT*-tag. Rh Bri2 BRICHOS has emerged as an interesting biopharmaceutical candidate for treating AD, as the domain is a potent inhibitor of Aβ42 amyloid fibril formation and in particular its associated neurotoxicity in vitro, in hippocampal slice preparations ex vivo*,* and in *Drosophila* models in vivo [15]*.* In addition, rh Bri2 BRICHOS passes the blood-brain barrier [16].

To increase the expression yield of rh Bri2 BRICHOS compared to standard shake flask cultivations, we reasoned that the medium composition and feeding with a suitable carbon source are key elements to obtain a high cell density culture, in addition to proper oxygen supply and pH control. Accordingly, three previously suggested medium formulations that are supposed to support cell densities higher than 70 g/L (dry cell weight) were tested for culturing *E. coli* SHuffle cells in a bioreactor using a fed-batch setup [26–28]. Interestingly, this initial experiment revealed that an OD_600_ above 100 was only obtained for the medium formulation described by da Silva et al*.,* why we chose this medium for all subsequent cultivations.

Furthermore, the influence of different IPTG concentrations for induction on the cell density at harvest and the expression level was tested (Table 1, culture #3, 6, 7, and 8). We observed a fourfold increase in expression level as the IPTG concentration was increased from 33 to 50 µM, however, the cell density at harvest was severely decreased if the IPTG concentration was 150 µM or higher, but without elevating the expression level. This confirms the importance of reducing and optimizing the inducer concentrations, to minimize cellular stress factors, as previously suggested for improved expression levels of soluble protein [28, 32]. Induction with 150 µM IPTG at OD_600_ of 30 resulted in an NT*-Bri2 BRICHOS expression level of 7.9 g/L. A minimal adjustment of this protocol by inducing protein expression at OD_600_ of 60 instead, elevated the expression level to 18.8 g/L (15.2 g/L if estimated from the dry cell weight), which competes with the highest reported recombinant protein expression levels for *E. coli* (Table 2) [26, 28, 37–44]. The caveat with this protocol is the high batch-to-batch variation, as repeating the exact cultivation conditions of #9 (culture #10 and #11) has not consistently resulted in expression levels of > 15 g/L. Nevertheless, at least 6 g/L NT*-Bri2 BRICHOS can be expected (Additional file 1: Table S1).

**Table 2:** Summary of some of the highest reported expression levels of recombinant protein using *E. coli*

Protein	Molecular Weight (kDa)	Expression level (g/L)	Yield after purification (g/L)	Solubility^a^	References
surface protein A from *Erysipelothrix rhusiopathiae*	42	6.4	n.r	n. r	da Silva et al. [26]
tilapia insulin-like growth factor-2	7	9.7	1.99^e^	5 M urea (IB)	Hu et al. [37]
human soluble B lymphocyte stimulator	18	3.8	n. r	n. r	Zhang et al. [39]
model cytoplasmic protein^b^	n. r	17.6^c^	n.r	0.1 M Tris–HCl	Kopp et al. [40]
chemotaxis protein CheY fused to green fluorescent protein from *Aequorea victoria*	n.r	12.0^d^	n.r	Bug Buster reagent	Wyre et al. [28]
human leptin	16	9.7	3.98^e^	8 M urea (IB)	Jeong et al. [41]
human interleukin 6	n.r	8.5	n.r	n. r	Tae et al. [38]
phenylalanine dehydrogenase mutant from*Tramitichromis intermedius*	40	n.r	4.6	20 mM KP_i_	Zhao et al. [42]
NT*-Bri2 BRICHOS	**29.6**	**18.8**	**6.5**	**20 mM Tris- HCl**	**This study**

n.r. – not reported. IB – inclusion bodies

^a^Refers to the buffer used to recover the proteins after cell lysis

^b^The exact identity of the model cytoplasmic protein is not reported

^c^Kopp et al. describe a repetitive fed-batch protocol, in which the expression level is increased after every round. The value here represents the expression level after the first round of fed-batch to make it comparable to the other values reported here. The highest expression level that Kopp et al. reported was 35.5 g/L

^d^Highest expression level reported. The highest concentration of soluble protein in the same study was 6 g/L

^e^Estimated from the reported % recovery after purification

Purification of Bri2-BRICHOS was executed in the same manner as previously described [25], and involved isolation of NT*-Bri2 BRICHOS with IMAC. The highest obtained yield after purification was 6.7 g/L (culture #9), which corresponds to 34% of the expressed protein. The main problem we faced during purification was that a large portion of NT*-Bri2 BRICHOS was found in the pellet after cell lysis/centrifugation and that the protein did not fully bind to the column and was to a large extent present in the flow-through (Fig. 1c). Therefore, the flow-through was reapplied to increase the yield, but still, a significant fraction was not recovered, which means that the yield most likely can be improved by additional optimizations.

Another relevant question that we wanted to investigate was whether the protein aggregation inhibition profiles of bioreactor produced rh Bri2 BRICHOS are comparable to previously published data for the shake flask-produced protein. To answer this, after removal of the NT*-tag with thrombin, rh Bri2 BRICHOS was separated into the individual oligomeric species using SEC. Interestingly, we were not only able to isolate monomers, dimers, and higher order oligomers but also tetramers, a species that we were not able to purify from our previous shake-flask preparations. The tetramer was predominantly formed after the removal of NT* from the NT*-Bri2 BRICHOS dimer. So far, we do not know what can trigger the transformation of the tetramer from the dimer, but the high concentration is probably an explanation as the Bri2 BRICHOS oligomerization process is sensitive to increased concentration [31]. Also, we cannot exclude the possibility that the composition of the cultivation medium, *e.g.* metals, could have potential effects on the equilibrium of the different species, and future work is necessary to elucidate the exact mechanisms behind this behavior. Nevertheless, in accordance with earlier results, CS aggregation was only inhibited by the presence of Bri2 BRICHOS oligomers. Likewise, the inhibition profile of Bri2 BRICHOS against Aβ42 amyloid formation is comparable, suggesting that the chaperoning capacities against fibrillar amyloid aggregation and non-fibrillar amorphous aggregation of Bri2 BRICHOS are independent of the expression and purification protocol.

## Conclusion

In this study, we establish an expression protocol of the human small heat shock protein chaperone domain Bri2 BRICHOS. Optimization of the process resulted in an expression level of up to 18.8 g/L, which is among the highest protein expression levels reported for *E. coli.* The high yield is likely attributed to the use of the NT*-tag, which drastically increases the solubility and expression levels of certain peptides and proteins. Since rh Bri2 BRICHOS is a promising candidate for treating AD, the protocol described in this paper is important to enable future research and successful translation into clinical trials.

## Supplementary Information

**Additional file 1.** **Table S1.** Summary of key parameters for the expression and purification of  NT*-Bri2 BRICHOS. **Figure S1.** SDS PAGE of NT*-Bri2 BRICHOS IMAC eluates. **Figure S2-3.** Estimation of the NT*-Bri2 BRICHOS expression level using SDS-PAGE. **Figure S4.** Native PAGE and SDS-PAGE of rh Bri2 BRICHOS.

## Data Availability

The datasets used and/or analyzed during the current study are available from the corresponding author on reasonable request.
