# Supplementary material for: Expression of the human molecular chaperone domain Bri2 BRICHOS on a gram per liter scale with an E. coli fed-batch culture
Source: Microb Cell Fact. 2021 Jul 30;20:150. doi: 10.1186/s12934-021-01638-8 (PMC8325310; doi:10.1186/s12934-021-01638-8)
Supplement: Supplementary file 1 — Additional file 1. Table S1. Summary of key parameters for the expression and purification of NT*-Bri2 BRICHOS. Figure S1. SDS PAGE of NT*-Bri2 BRICHOS IMAC eluates. Figure S2-3. Estimation of the NT*-Bri2 BRICHOS expression level using SDS-PAGE. Figure S4. Native PAGE and SDS-PAGE of rh Bri2 BRICHOS. [file 12934_2021_1638_MOESM1_ESM.docx]

Additional file 1

**Expression of the human molecular chaperone domain Bri2 BRICHOS on a gram per liter scale with an *E. coli* fed-batch culture**

Benjamin Schmuck^1,2*^, Gefei Chen^1^, Josef Pelcman^1^, Nina Kronqvist^1^, Anna Rising^1,2^, and Jan Johansson^1^

1 Department of Biosciences and Nutrition, Karolinska Institutet, Neo, 141 86 Huddinge, Sweden

2 Department of Anatomy, Physiology and Biochemistry, Swedish University of Agricultural

Sciences, Uppsala, Sweden

*corresponding author: benjamin.schmuck@ki.se

| **Table S1.** Summary of key parameters for the expression and purification of NT*-Bri2 BRICHOS expressed in bioreactor cultivations #9-11. | | | |
| --- | --- | --- | --- |
| Culture | #9 | #10 | #11 |
| Temperature (°C) | 25 | 25 | 25 |
| Medium | da Silva | da Silva | da Silva |
| Substrate in feed | Glycerol | Glycerol | Glycerol |
| IPTG (µM) | 150 | 150 | 150 |
| OD_600_ induced | 60 | 59 | 72 |
| OD_600_ harvest | 176 | 132 | 126 |
| Induction time (h) | 22 | 22 | 22 |
| Total culture time (h) | 49 | 53 | 53 |
| Culture Size (mL) | 1180 | 1160 | 1150 |
| Wet Cell Weight (g/L) | 214 | 167 | 171 |
| Dry Cell Weight (g/L) | 82 | 55 | 48 |
| Expression level (g/L)^a^ | 18.8 | 10.3 | 6.5 |
| Dry cell: Expression level (g/L)^b^ | 15.2 | 9.6 | 6.4 |
| % of culture purified | 7.1% | 8.8% | 8.9% |
| Purified (mg)^c^ | 540 | 354 | 229 |
| Yield after purification (g/L)^d^ | 6.41 | 3.45 | 2.25 |
| % of expression level | 34.1% | 33.5% | 34.6% |
| a) Expression level of NT*-Bri2 BRICHOS was estimated with SDS-PAGE. b) The expression level calculated by considering the % of NT*-Bri2 BRICHOS relative to the total protein content and the dry cell mass. c) Protein yield was calculated using Abs_280_ and the protein specific extinction coefficient after NT*-Bri2 BRICHOS was eluted and dialyzed to remove imidazole. d) Yield of Bri2 BRICHOS after purification. | | | |

**Figure S1.** SDS-PAGE of NT*-Bri2 BRICHOS IMAC eluates expressed in bioreactor cultivations #1-11. Eluate 1 designates the protein that was obtained after the lysate was applied onto the column followed by wash and elution. The flow-through was reapplied onto the column in the same manner (eluate 2) to increase the yield of NT*-Bri2 BRICHOS.


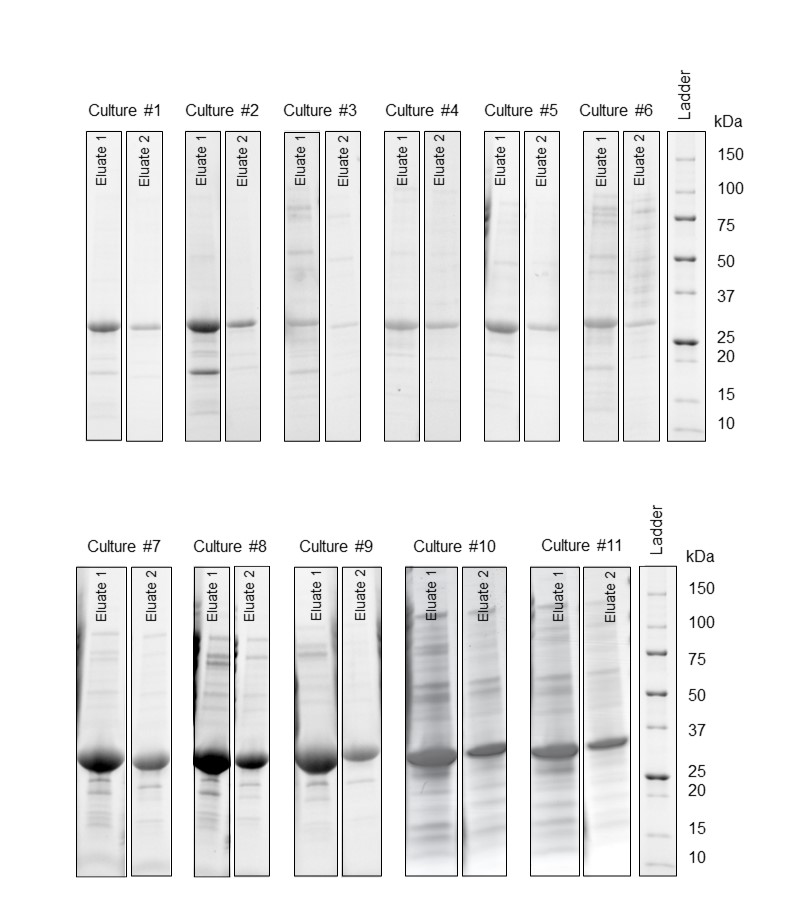


**Figure S2.** Estimation of the NT*-Bri2 BRICHOS expression level using SDS-PAGE (left). Already purified NT*-Bri2 BRICHOS was used to create a standard curve (middle). The standard curve was used to calculate the concentration of NT*-Bri2 BRICHOS in the bioreactor culture before harvest (right), also considering the dilution factor of the samples loaded onto the SDS-PAGE gel. (A) Culture #1 & 2. (B) Culture #3-5. (C) Culture #6-8.


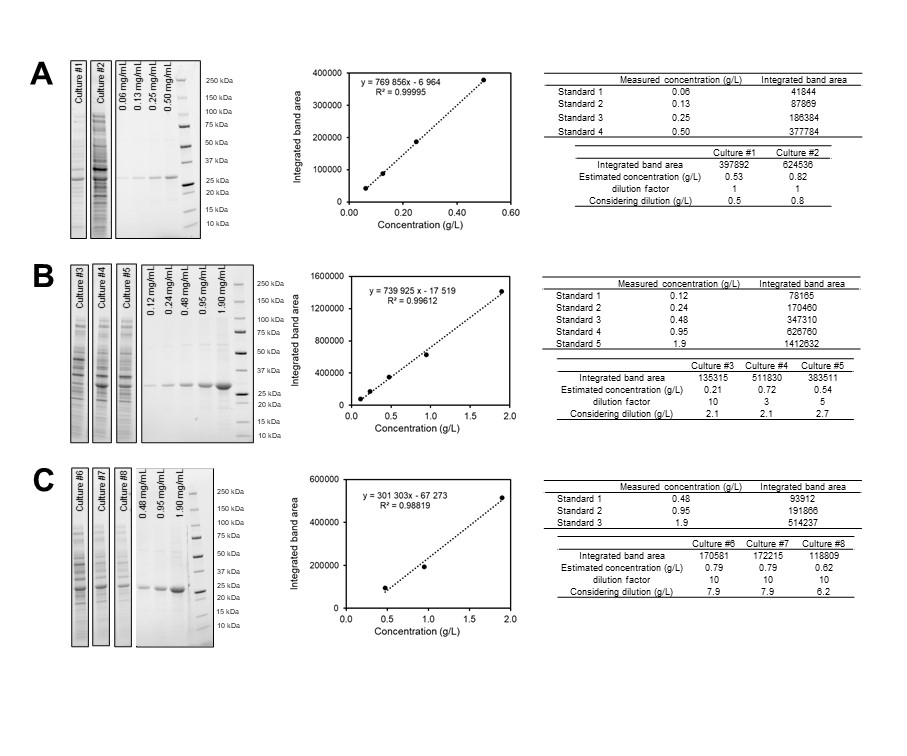

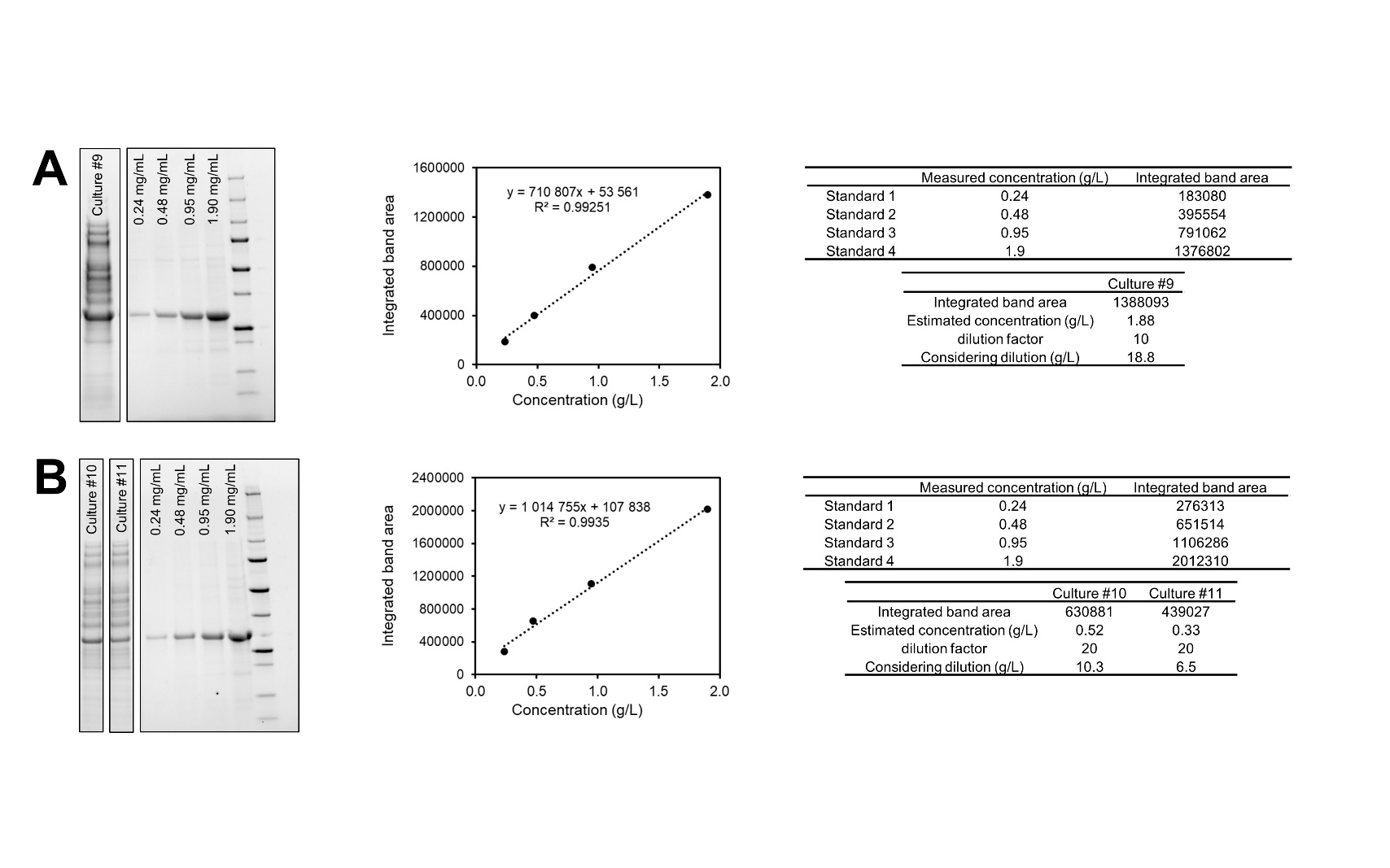


**Figure S3.** Estimation of the NT*-Bri2 BRICHOS expression level using SDS-PAGE (left). Already purified NT*-Bri2 BRICHOS was used to create a standard curve (middle). The standard curve was used to calculate the concentration of NT*-Bri2 BRICHOS in the bioreactor culture before harvest (right), also considering the dilution factor of the samples loaded onto the SDS-PAGE gel. (A) Culture #9 (B) Culture #10-11.


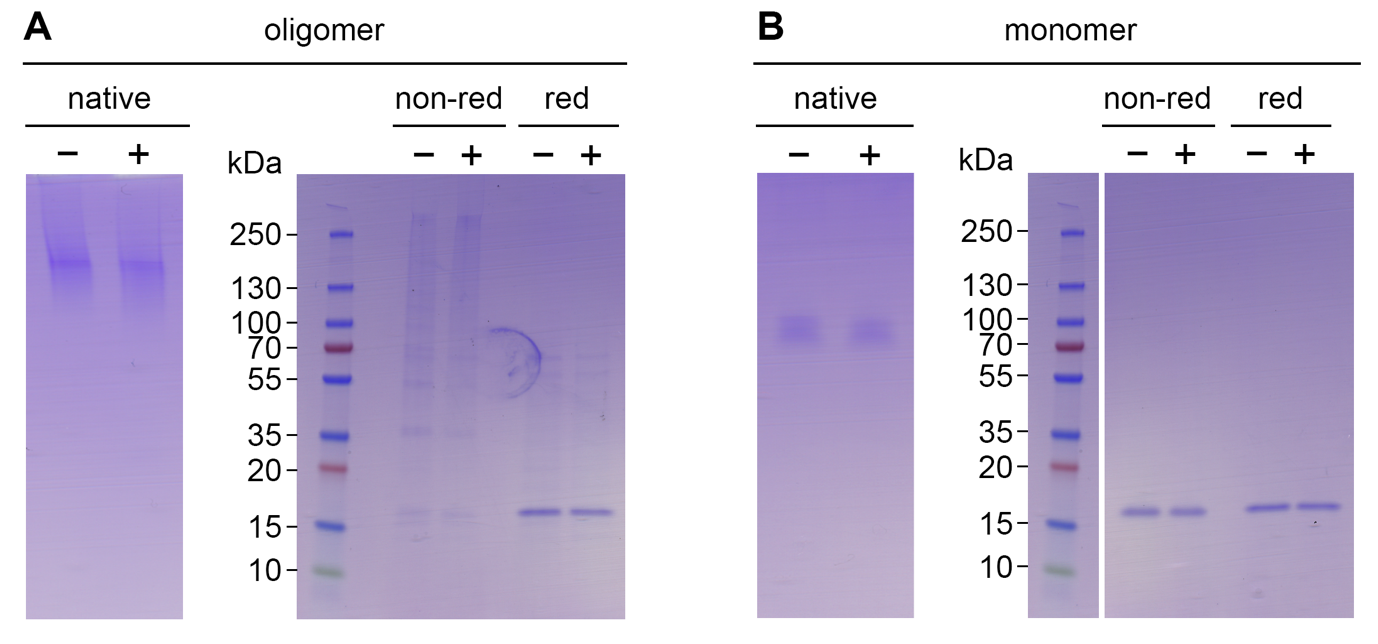


**Figure S4.** Native PAGE and SDS-PAGE of rh Bri2 BRICHOS oligomers (A) and monomers (B) before (-) and after (+) incubation at 37°C over night. The samples were loaded onto the SDS-PAGE gel using a non-reducing (non-red) or a reducing (red) loading buffer.
